# Correlation between the expression of LAT1 in cancer cells and the potential efficacy of boron neutron capture therapy

**DOI:** 10.1093/jrr/rrac077

**Published:** 2022-11-14

**Authors:** Tsubasa Watanabe, Yu Sanada, Yoshihide Hattori, Minoru Suzuki

**Affiliations:** Institute for Integrated Radiation and Nuclear Science, Kyoto University, Osaka, Japan; The Hakubi Project, Kyoto University, Kyoto, Japan; Institute for Integrated Radiation and Nuclear Science, Kyoto University, Osaka, Japan; Research Center for Boron Neutron Capture Therapy, Osaka Metropolitan University, 1-1 Gakuen-cho, Nakaku, Sakai, Osaka 599-8531, Japan; Institute for Integrated Radiation and Nuclear Science, Kyoto University, Osaka, Japan

**Keywords:** boron neutron capture therapy (BNCT), p-boronophenylalanine (BPA), Borofalan(^10^B), SLC7A5, LAT1

## Abstract

Boron neutron capture therapy (BNCT) is a binary cancer therapy that involves boron administration and neutron irradiation. The nuclear reaction caused by the interaction of boron atom and neutron produces heavy particles with highly cytocidal effects and destruct tumor cells, which uptake the boron drug. p-Boronophenylalanine (BPA), an amino acid derivative, is used in BNCT. Tumor cells with increased nutrient requirements take up more BPA than normal tissues via the enhanced expression of LAT1, an amino acid transporter. The current study aimed to assess the correlation between the expression of LAT1 and the uptake capacity of BPA using genetically modified LAT1-deficient/enhanced cell lines. We conducted an *in vitro* study, SCC7 tumor cells wherein LAT1 expression was altered using CRISPR/Cas9 were used to assess BPA uptake capacity. Data from The Cancer Genome Atlas (TCGA) were used to examine the expression status of LAT1 in human tumor tissues, the potential impact of LAT1 expression on cancer prognosis and the potential cancer indications for BPA-based BNCT. We discovered that the strength of LAT1 expression strongly affected the BPA uptake ability of tumor cells. Among the histologic types, squamous cell carcinomas express high levels of LAT1 regardless of the primary tumor site. The higher LAT1 expression in tumors was associated with a higher expression of cell proliferation markers and poorer patient prognosis. Considering that BPA concentrate more in tumors with high LAT1 expression, the results suggest that BNCT is effective for cancers having poor prognosis with higher proliferative potential and nutritional requirements.

## INTRODUCTION

Boron neutron capture therapy (BNCT) is a binary cancer therapy that involves boron administration and neutron irradiation, which result in the interaction of boron atom (^10^B) and neutron [1,2]. When ^10^B captures a low-energy neutron, the interaction of ^10^B and neutron leads to a nuclear fission reaction and produces heavy particles (an alpha particle and a lithium nucleus) with a highly cytocidal effect. This mechanism is referred to as the boron neutron capture reaction. The flight range of these particles in water is < 10 (range: 5–9) μm, which is shorter than the diameter of a single cell. Therefore, if a sufficient amount of boron atoms accumulates in tumor cells, neutron irradiation can selectively destruct tumor cells using the fission reaction of boron atoms within these cells.

The anti-tumor effect of BNCT is via the selective delivery of boron atoms to tumor cells through boron drugs [2,3]. Currently, p-boronophenylalanine (BPA, borofalan(^10^B)) is the most effective agent used in BNCT for cancer [4]. Based on clinical trials, BPA-based BNCT has been established as safe and effective for cancer treatment and is covered by insurance for the treatment of unresectable locally advanced or locally recurrent head and neck cancers in Japan [5]. BPA was first used as a boron drug for BNCT in 1986 [6]. Thereafter, various efforts have been made to enhance the therapeutic efficacy of BPA-based BNCT. These include improving drug solubility and optimizing administration methods [4–8]. In principle, BNCT is a unique therapy that can selectively deliver heavy ion beams at the cellular level. Moreover, its features, such as the capability to treat recurrence at sites where radiation has already been delivered relatively safely, are not found in other radiation therapies.

Expanding the clinical indications of BNCT to cancers other than head and neck cancer is a future challenge. The structure of BPA is similar to that of phenylalanine and tyrosine. The latter are used as raw materials for the biosynthesis of melanin. Therefore, BPA was first developed as a tumor cell-selective boron agent for melanin-bearing tumors and malignant melanoma [6,9]. Subsequently, BPA was found to be actively taken up by other tumors with increased nutrient requirements [10]. The efficacy and safety of BPA-based BNCT is currently evaluated in clinical trials on malignant melanoma, malignant brain tumors and hemangiosarcoma. The use of clinical data and the identification of tumors with a propensity to take up BPA selectively are essential in detecting tumor candidates for BPA-based BNCT in the future.

Among several amino acid transporters in cells, SLC7A5 (L-type amino acid transporter 1, LAT1), a neutral amino acid transporter that is highly expressed in tumor cells, is mainly responsible for BPA uptake [11–13]. However, only a few reports have directly analyzed the association between the strength of LAT1 expression and BPA uptake in tumor cells. In addition, to date, no report has examined the expression of LAT1 in different types of cancers using human clinical specimens in terms of BPA-based BNCT. If there is an evident association between the strength of LAT1 expression in tumor cells and BPA uptake, the expression of LAT1 in human clinical specimens can provide a reference in determining the indications for BPA-based BNCT. The current study first assessed the effect of LAT1 expression on BPA uptake in tumor cells with genetically enhanced or inhibited LAT1 expression. Further, a group comprising diseases that can be potential candidates for the future expansion of BPA-based BNCT indications, with a focus on the expression of LAT1, were analyzed using data from a cancer database.

## MATERIALS AND METHODS

### Materials

This study used cell lines generated from the SCC7 tumor cell line, a murine squamous cell carcinoma cell line that obtained spontaneously from a C3H/He mouse (Department of Radiation Oncology and Image-Applied Therapy, Kyoto University, Kyoto, Japan). SCC7 cells expressing the 6 × His-tagged LAT1 protein (SCC7-WT) were previously established [14], and the LAT1-deficient (SCC7-ΔLAT1) and LAT1-overexpressing (SCC7-LAT1_enhance) cells were established from the SCC7-WT cells, as described below. These cells were maintained in Dulbecco’s modified Eagle medium supplemented with 10% fetal bovine serum and penicillin/streptomycin (100 U/mL). The cells were cultured at 37°C in a 5% CO_2_ incubator. BPA was purchased from Interpharma (Prague, Czech Republic). 2-Aminobicyclo-(2.2.1)-heptane-2-carboxylic acid (BCH), an inhibitor of system L amino acid transporters, was purchased from Cayman Chemical (Michigan, USA). BPA was used in the aqueous solution as a fructose complex [15,16]. Briefly, BPA, fructose and 1 N NaOH were dissolved in distilled water at a molar ratio of 1:1.5:1.15. The mixture was stirred until BPA had dissolved completely, and the pH value was titrated to 7.6 with 1 N HCl. The solution was filtered using a 0.22-μm syringe filter for sterilization (Merck Millipore, Massachusetts, USA).

### Establishment of LAT1-deficient and LAT1-overexpressing cells

For SCC7-ΔLAT1, CRISPR/Cas9 expression vector #1 for the *Slc7a5* locus was used (pX330, Addgene #42230). CCGGACTGTCGCTCGTGGTG(TGG) was the guide sequence for mouse *Slc7a5* targeting. SCC7-WT was transfected with CRISPR/Cas9 expression vector #1 using Lipofectamine 3000 (Invitrogen, Massachusetts, USA), and the single cell clones of genome-modified cells were obtained by limiting dilutions. Genomic regions surrounding the target site were sequenced and compared to the wild-type, as previously described [17]. The genomes of the transfected cells used in this study had insertion–deletion mutations (indels) in the target sequences. All detected indels were predicted to change the translational reading frame. For SCC7-LAT1_enhance, we designed a targeting vector containing a CMV promoter, mouse LAT1 cDNA fragment fused to the His-tag sequence and a neo-resistance marker. CRISPR/Cas9 expression vector #2 for the *Rosa26* locus was generated using pX330. ACTCCAGTCTTTCTAGAAGA(TGG) was the guide sequence for mouse *Rosa26* targeting [18]. SCC7-WT cells were transfected with targeting vector #2 and CRISPR/Cas9 expression vector. G418 at dose of 0.5 mg/mL was added to the media 48 h after transfection for selection, and the G418-resistant clones were obtained. The expression of LAT1 in these transfected cells were confirmed via Western blot analysis using an antibody against 6 × His-tag (Bethyl Laboratories, 1:1000). The quantification cycle (C_q_) value of LAT1 and beta actin mRNA were measured by reverse transcription-polymerase chain reaction (RT-PCR) (MyGo Mini S, IT-IS International Ltd., Middlesbrough, UK). Beta actin was used as a reference for calculating the ΔΔC_q_ value of LAT1 in RT-PCR.

### Measurements of boron concentration in the cells

After the incubation of the tumor cells (2.0 × 10^5^) with 1 mM BPA for 30 min, cells were rapidly washed twice with cold (4°C) Hanks’ Balanced Salt Solution without Ca^2+^ and Mg^2+^, and digested with perchloric acid (60%) and hydrogen peroxide (30%) for 3 h at 75°C. In the BCH-treated group, cells were pre-treated with 1 mM BCH before BPA administration for 10 min. After the pre-treatment, cells were incubated with medium containing 1 mM BPA and 1 mM BCH. The boron concentration of the cell lysate was assessed via inductively coupled plasma atomic emission spectrometry (ICP-AES) (ICPE-9000, Shimazu, Shimazu, Tokyo, Japan) and was normalized as ^10^B μg per 10^7^ cells.

**Fig. 1:**
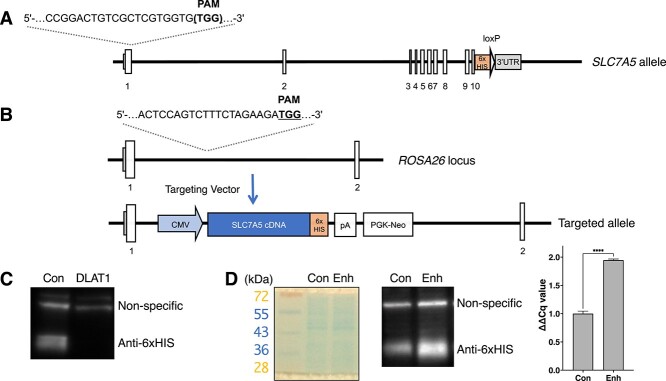
The expression status of LAT1 in the cells used in this study. (A) Schematic diagram of the *Slc7a5* locus, 6 × His-tag, and the targeted site of CRISPR/Cas9 for the generation of SCC7-ΔLAT1. (B) Schematic diagram of the *Rosa26* locus for the generation of SCC7_LAT1_enhance. (C) Immunoblot analysis of SCC7-ΔLAT1. Con (control): SCC-WT cells before the generation of SCC7-ΔLAT1 cells by CRISPR/Cas9, DLAT1: SCC7-ΔLAT1 cells. (D) CBB stain, immunoblot and RT-PCR results of SCC7_LAT1_enhance. Con (control): SCC-WT cells transfected with an empty vector containing only a neomycin-resistance cassette (PGK-neo), Enh: SCC7_LAT1_enhance cells. A *P* value of less than 0.05 were considered to be statistically significant (^****^P < 0.0001).

**Fig. 2:**
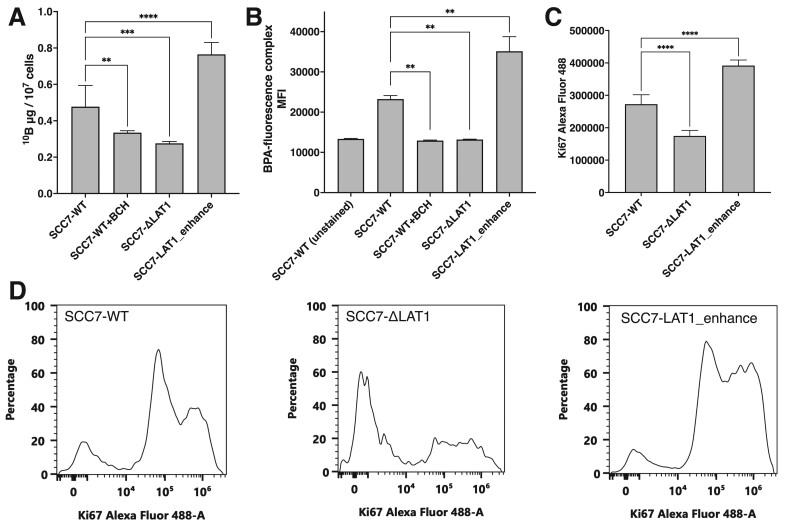
p-Boronophenylalanine (BPA) uptake capacity based on the expression of LAT1 in cancer cells. (A) Boron concentration in tumor cells with genetically modified tumor cells assessed via inductively coupled plasma atomic emission spectrometry (*n* = 6). Values were normalized as ^10^B μg per 10^7^ cells. (B) MFI of the complex of BPA and BPA-probe in tumor cells evaluated via flow cytometer (*n* = 4). (C) Mean Alexa Fluor 488 fluorescent intensity of Ki67 evaluated via flow cytometer (*n* = 5). (D) Representative histogram of the Ki67 fluorescent intensity of SCC7-WT, SCC7-ΔLAT1 and SCC7-LAT1_enhance. *P*-values of < 0.05 were considered to be statistically significant (^**^P < 0.01, ^***^P < 0.001, ^****^P < 0.0001).

### Flow cytometry

The BPA uptake capacity of tumor cells was assessed using the amino acid uptake assay kit (#UP04–12, DOJINDO Laboratories, Kumamoto, Japan). Briefly, BPA was used as amino acid analogs, and the BPA uptake capacity was assessed using BPA-probe, which penetrates the cell membrane, binds to the intracellular BPA and emits strong fluorescence if bound to BPA [19–21]. The median fluorescent intensity (MFI) of the complex of BPA with BPA-probe in the cells was assessed using Cytek NL2000 full spectrum flow cytometer (Cytek Japan, Tokyo, Japan). For the measurement of Ki67 protein expression intensity, cells were measured with the same cytometer using Alexa Fluor 488 anti-mouse/human Ki-67 antibody (clone 11F6) (BioLegend, San Diego, USA) and Zombie Red fixable viability dye (BioLegend, San Diego, USA) per the manufacturer’s Ki67-staining protocol.

### Analyses of data from a human cancer database

Data on patients with cancer were extracted from The Cancer Genome Atlas (TCGA) (TCGA38) with QIAGEN OmicSoft ArrayStudio (QIAGEN, Veno, The Netherlands). Patients were categorized according to the expression of LAT1 (high = top 1/2 of LAT1 expression levels, low = lower 1/2 of LAT1 expression levels), and the overall survival curve was compared based on the LAT1 expression categories. The extent of LAT1 in different types of cancer was assessed using the value of fragments per kilobase million (FPKM). The correlation between the expression levels of LAT1 and Ki67 and SLC6A14, another amino acid transporter, was compared among tumor types (head and neck cancer, melanoma and glioblastoma). Differential gene expression between tumor and normal tissues were analyzed using TIMER2.0 [22–24].

### Statistical analysis

Data were expressed as the mean ± standard deviation. Between groups differences in *in vitro* experiments were analyzed using t-test (RT-PCR) or one-way analysis of variance with Dunnett’s multiple comparison correction (ICP–AES and flow cytometry). The Kaplan–Meier method was used to compare survival curves between groups. A *P*-value (two-sided tests) of < 0.05 were considered statistically significant (ns = *P* ≥ 0.05, ^*^P < 0.05, ^**^P < 0.01, ^***^P < 0.001, ^****^P < 0.0001). All statistical analyses were performed using Prism 9 (GraphPad Software, San Diego, CA, USA).

## RESULTS

### Importance of LAT1 expression in cancer cells on BPA uptake capacity

To examine the association between the expression of LAT1 and BPA uptake, LAT1-deficient (SCC7-ΔLAT1) and LAT1-overexpressing (SCC7-LAT1_enhance) cells were established from the SCC7-WT cells. Figure 1A shows the *Slc7a5* locus of SCC7-WT cells, which express 6 × HIS-tagged LAT1 and are the targeted site for SCC7-ΔLAT1 generation. Figure 1B shows the *ROSA26* locus of SCC7-LAT1_enhance. As shown in Figs 1C and1D, SCC7-ΔLAT1 and SCC7-LAT1_enhance cells have low and high expressions of LAT1 compared to SCC7-WT cells, respectively. The RT-PCR results showed that SCC7 cells had 1.95-fold higher LAT1 expression relative to beta actin than the control group (Fig. 1D). Figure 2A shows the BPA uptake in these cells evaluated via ICP–AES. Treatment with BCH, a LAT1 inhibitor, significantly suppressed BPA uptake. Similarly, LAT1-deficient cells in the BCH-treated group had reduced BPA uptake capacity. Further, BPA uptake was amplified in tumor cells with a higher LAT1 expression. Figure 2B shows the BPA uptake in tumor cells assessed via flow cytometry using a boron probe that binds to BPA. The fluorescence intensity of tumor cells with enhanced LAT1 expression significantly increased. This information was consistent with data obtained via ICP–AES.

### LAT1 expression levels were correlated with Ki67 expression signals measured by flow cytometry *in vitro*

Figure 2C shows the mean fluorescent intensity of Ki67 for SCC7-WT, SCC7-ΔLAT1 and SCC7-LAT1_enhance cells, respectively. Figure 2D shows a typical histogram of Ki67 measured by flow cytometry, showing that SCC7-ΔLAT1 has majority of cells with little or no Ki67 expression, whereas SCC7-LAT1_enhance has a large percentage of cells with strong Ki67 expression. The intensity of LAT1 expression in tumor cells also significantly changed the intensity of Ki67 expression, with SCC7-ΔLAT1 having significantly weaker Ki67 expression and SCC7-LAT1_enhance having significantly stronger Ki67 expression compared to SCC7-WT.

### Potential human cancer candidates for BPA-based BNCT

**Fig. 3:**
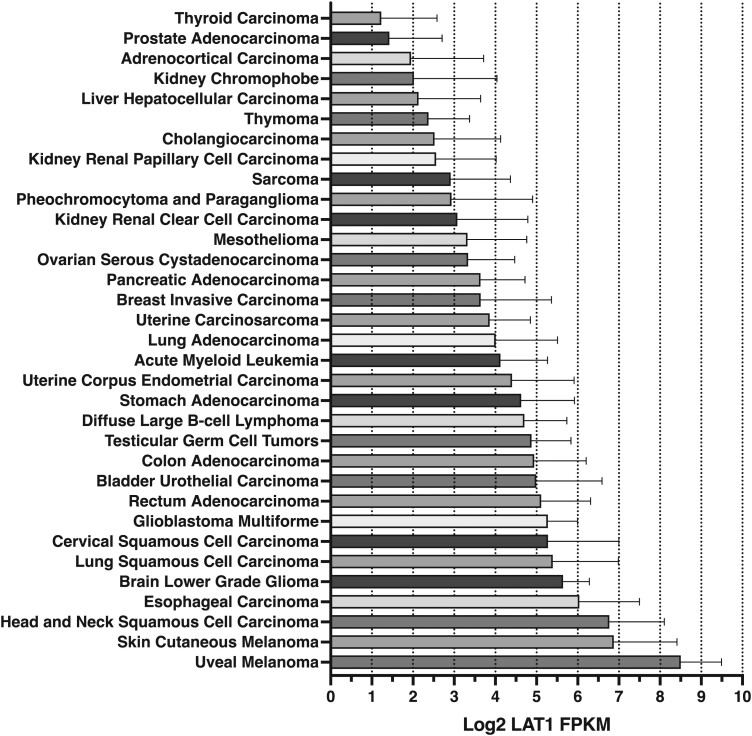
Expression of LAT1 in human cancer. Bars are expressed as the mean ± SD. FPKM, the value of fragments per kilobase million.

Figure 3 shows the LAT1 expression in different types of cancers based on data collected from TCGA. The horizontal axis, as depicted in Fig. 3, indicated the intensity of LAT1 expression. Unlike other tumors, malignant melanoma had a high expression of LAT1. According to histological type, squamous cell carcinoma was the most common tumor with a high expression of LAT1. In addition to head and neck tumors, esophageal and cervical tumors and squamous cell carcinoma of the lung have a high expression of LAT1 (Fig. 3). Further, a relatively high expression of LAT1 was observed in transitional epithelial carcinoma, which is the primary histological type of bladder and urinary tract cancer. The strength of LAT1 expression in tumors with adenocarcinoma histology (such as gastrointestinal, breast and uterine cancer and lung adenocarcinoma) and lymphomas was similar to that in glioblastoma.

### Correlation between the expression of LAT1 and poor prognosis

**Fig. 4:**
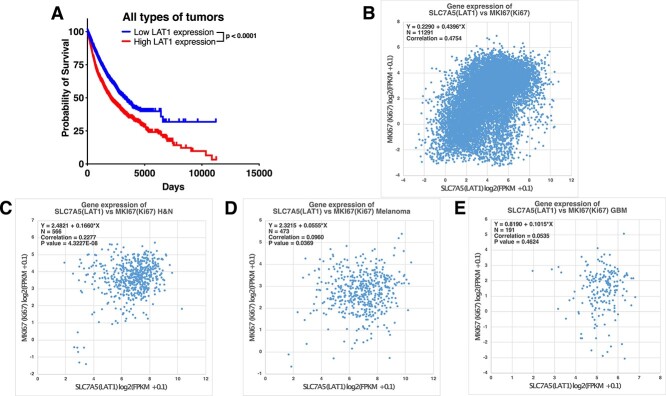
Correlation between high LAT1 expression in tumor tissues and poor prognosis. (A) Survival curves of patients with cancer in TCGA stratified according to LAT1 expression. (B) Two-dimensional plots of SLC7A5 (LAT1) and MKI67 (Ki67) in all types of tumors. (C) Two-dimensional plots of SLC7A5 (LAT1) and MKI67 (Ki67) in patients with head and neck cancer (H&N). (D) Two-dimensional plots of SLC7A5 (LAT1) and MKI67 (Ki67) in patients with melanoma. (E) Two-dimensional plots of SLC7A5 (LAT1) and MKI67 (Ki67) in patients with glioblastoma (GBM). FPKM, the value of fragments per kilobase million.

Figure 4A shows the survival curves of patients with all types of cancer in TCGA, which were stratified according to the strength of LAT1 expression. Based on the survival curves of all patients with cancer based on the expression of LAT1, patients with cancer who had a low LAT1 expression in the tumor tissue were more likely to have a better prognosis than those with high LAT1 expression. A similar trend was observed in head and neck cancer and melanoma (Supplementary Fig. 1A–1B). There was no evident difference in the survival curves of patients with malignant brain tumors based on the expression of LAT1 (Supplementary Fig. 1C).

A significant positive correlation was observed between the expression of LAT1 and Ki67. However, there was no evident correlation between LAT1 and another amino acid transporter, SLC6A14 (ATB^0,+^). The expression of SLC6A14 in all types of cancer tissues was more likely to be classified as minimal or no expression and strong expression (Supplementary Fig. 2A–2D). Differential gene expression between tumor and corresponding normal tissues indicated that the tumor tissues had a higher expression of LAT1 than the corresponding normal tissues (Supplementary Fig. 3). The expression of respective transporters in tumor and normal tissues was not as relevant as that of LAT1 (Supplementary Fig. 3B–C). The presence or absence of SLC6A14 expression varied significantly among different tumor types (Supplementary Fig. 3D).

## DISCUSSION

The expansion of BNCT indications is an essential aspect in the future development of BNCT. The current study used data from a human cancer database, and results showed a trend in LAT1 expression based on cancer histology. In particular, the expression of LAT1 was more likely to be high in squamous cell carcinoma. In addition to head and neck cancers, esophageal and cervical cancers, particularly squamous cell carcinomas, may have a higher BPA uptake capacity than other tumors. Lung cancer is among the most common types of cancers worldwide. Lung squamous cell carcinoma accounts for approximately one-fourth of all lung cancer cases [25]. Squamous cell carcinoma of the lung is associated with a high LAT1 expression, and BPA-based BNCT may be effective against squamous cell carcinoma of the lung if a sufficient amount of neutrons can be irradiated to the tumor. Moreover, a relatively high LAT1 expression was observed in transitional epithelial bladder and urinary tract cancers. Surprisingly, the LAT1 expression level of tumors with adenocarcinoma histology and lymphomas was similar to that of glioblastoma. Considering the efficacy of BPA-based BNCT against glioblastoma [26,27], patients with adenocarcinomas may also benefit from such treatment.

We found that higher LAT1 expression tends to have a significantly worse prognosis in all types of tumor (Fig. 4A), head and neck cancer and melanoma (Supplementary Fig. 1). Especially in glioblastoma, we did not find significant differences in survival curves and LAT1 expression. Figure 3 shows that for brain tumors, benign brain tumors tend to have higher LAT1 expression than malignant brain tumors. The inverse correlation between LAT1 expression and grade may be responsible for the lack of correlation between LAT1 expression and prognosis in glioblastoma.

When expanding the indications for BPA-based BNCT, attention should be paid to the BPA uptake capacity of normal tissues at the neutron irradiation site. BPA is a derivative of amino acids, and normal tissues take up BPA at a lesser extent than cancerous tissues [25]. In particular, in terms of abdominal organs, the pancreas is a potential uptake site for BPA, and other abdominal organs such as the intestinal tract and liver may also take up BPA, but not as much as tumor tissues [28]. In relation to this, BPA-based BNCT should be applied in the treatment of gastrointestinal cancers only if its safety has been validated. The authors have proposed a tumor-to-normal tissue ratio (T/N) enhancer strategy. That is, T/N enhancer is pre-administered prior to BPA administration. T/N enhancers are amino acids or amino acid derivatives that reduce the BPA uptake in normal tissues ideally without changing the BPA uptake capacity of tumor tissues [29]. BPA-based BNCT may be safer for gastrointestinal cancers particularly if T/N enhancers, which can inhibit BPA uptake in the intestinal tract, liver and pancreas, can be developed in the future.

In this study, we assessed the effect of LAT1 expression on BPA uptake in tumor cells with genetically enhanced or inhibited LAT1 expression. In this study, no experiments were conducted to irradiate tumors with neutrons. In a previous study, we and our colleagues have shown that BPA-based BNCT could destruct cancer cells by enhancing the expression of LAT1 after neutron irradiation [30]. This finding is also consistent with the current our results on SCC7-LAT1_enhance cells. In addition, we have shown that genetically modified cells with low expression of LAT1 uptake less BPA compared to control and a group with a LAT1 inhibitor. Taken together, these data support the hypothesis that the expression of LAT1 in cancer cells is associated with BPA uptake capacity, and the extent of LAT1 expression can be a surrogate marker of BPA uptake in tumor tissues.

The expression of LAT1 in tumor tissues was found to be correlated with cell proliferation markers both *in vitro* (Fig. 2) and in human participants (Fig. 4) in this study. Moreover, patients with cancer which have a high LAT1 expression may have a poor prognosis (Fig. 4 and Supplementary Fig. 1). BPA-based BNCT is more effective in treating tumors with high LAT1 expression. Therefore, it can be useful in treating cancers with a poor prognosis. This study first elucidated this unique feature of BNCT not found in other therapies.

Notably, the intensity of LAT1 expression in tumor cells is extremely different from that in a proportion of cells in tissue samples evaluated via immunohistochemistry [31], which uses agents such as horseradish peroxidase and enzymatically amplifies the LAT1 expression signal. This enzymatic amplification of the signal is effective in assessing the presence or absence of protein expression, but not its strength. This might be the reason why BPA uptake could not be accurately quantified based on the proportion of LAT1 expression area via immunohistochemistry [31]. If the current study results will be used as a reference to determine the indications for BNCT clinically, the intensity of LAT1 expression should be identified using methods that do not amplify the LAT1 expression signals or those that properly correct LAT1 protein expression signals.

## CONCLUSION

The expression of LAT1 is important for BPA uptake, and BPA-based BNCT may be used for the treatment of squamous cell carcinoma and transitional epithelial carcinoma via the enhanced expression of LAT1. Moreover, adenocarcinomas, lymphomas, glioblastoma had a similar expression of LAT1. Hence, the study results can provide important guidelines for the future expansion of BNCT indications.

## Supplementary Material

Suppl_Fig1_modifiedrrac077

Suppl_Fig2_rrac077

Suppl_Fig3_rrac077

Supplementary_data_rrac077

## References

[1] Barth RF, Coderre JA, Vicente MGH et al. Boron neutron capture therapy of cancer: current status and future prospects. Clin Cancer Res 2005;11:3987–4002. 15930333 10.1158/1078-0432.CCR-05-0035

[2] Barth RF, Vicente MGH, Harling OK et al. Current status of boron neutron capture therapy of high grade gliomas and recurrent head and neck cancer. Radiat Oncol 2012;7:146–66. 22929110 10.1186/1748-717X-7-146 PMC3583064

[3] Barth RF, Mi P, Yang W. Boron delivery agents for neutron capture therapy of cancer. Cancer Commun 2018;38:35–49. 10.1186/s40880-018-0299-7 PMC6006782 29914561

[4] Hiratsuka J, Kamitani N, Tanaka R et al. Long-term outcome of cutaneous melanoma patients treated with boron neutron capture therapy (BNCT). J Radiat Res 2020;61:945–51. 32990318 10.1093/jrr/rraa068 PMC7674695

[5] Hirose K, Konno A, Hiratsuka J et al. Boron neutron capture therapy using cyclotron-based epithermal neutron source and borofalan (^10^B) for recurrent or locally advanced head and neck cancer (JHN002): an open-label phase II trial. Radiother Oncol 2021;155:182–7. 33186684 10.1016/j.radonc.2020.11.001

[6] Mishima Y, Honda C, Ichihashi M et al. Treatment of malignant melanoma by single thermal neutron capture therapy with melanoma-seeking 10B-compound. Lancet 1989;2:388–9. 2569577 10.1016/s0140-6736(89)90567-9

[7] Fukuda H, Kobayashi T, Matsuzawa T et al. RBE of a thermal neutron beam and the 10B(n, alpha)7Li reaction on cultured B-16 melanoma cells. Int J Radiat Biol Relat Stud Phys Chem Med 1987;51:167–75. 3492464 10.1080/09553008714550601

[8] Yoshino K, Suzuki A, Mori Y et al. Improvement of solubility of p-boronophenylalanine by complex formation with monosaccharides. Strahlenther Onkol 1989;165:127–9. 2928932

[9] Coderre JA, Glass JD, Fairchild RG et al. Selective targeting of boronophenylalanine to melanoma in BALB/c mice for neutron capture therapy. Cancer Res 1987;47:6377–83. 3677082

[10] Coderre JA, Glass JD, Fairchild RG et al. Selective delivery of boron by the melanin precursor analogue p-boronophenylalanine to tumors other than melanoma. Cancer Res 1990;50:138–41. 2293547

[11] Detta A, Cruickshank GS. L-amino acid transporter-1 and boronophenylalanine-based boron neutron capture therapy of human brain tumors. Cancer Res 2009;69:2126–32. 19244126 10.1158/0008-5472.CAN-08-2345

[12] Wongthai P, Hagiwara K, Miyoshi Y et al. Boronophenylalanine, a boron delivery agent for boron neutron capture therapy, is transported by ATB ^0,+^, LAT 1 and LAT 2. Cancer Sci 2015;106:279–86. 25580517 10.1111/cas.12602 PMC4376436

[13] Yoshimoto M, Kurihara H, Honda N et al. Predominant contribution of L-type amino acid transporter to 4-borono-2-(18)F-fluoro-phenylalanine uptake in human glioblastoma cells. Nucl Med Biol 2013;40:625–9. 23557719 10.1016/j.nucmedbio.2013.02.010

[14] Sanada Y, Takata T, Tanaka H et al. HIF-1α affects sensitivity of murine squamous cell carcinoma to boron neutron capture therapy with BPA. Int J Radiat Biol 2021;97:1441–9. 34264166 10.1080/09553002.2021.1956004

[15] Mori Y, Suzuki A, Yoshino K et al. Complex formation of p-boronophenylalanine with some monosaccharides. Pigment Cell Res 1989;2:273–7. 2508079 10.1111/j.1600-0749.1989.tb00203.x

[16] Watanabe T, Hattori Y, Ohta Y et al. Comparison of the pharmacokinetics between L-BPA and L-FBPA using the same administration dose and protocol: a validation study for the theranostic approach using [18F]-L-FBPA positron emission tomography in boron neutron capture therapy. BMC Cancer 2016;16:859–68. 27821116 10.1186/s12885-016-2913-x PMC5100278

[17] Sanada Y, Sasanuma H, Takeda S et al. Disruption of Hif-1α enhances cytotoxic effects of metformin in murine squamous cell carcinoma. Int J Radiat Biol 2018;94:88–96. 29185833 10.1080/09553002.2018.1409443

[18] Chu VT, Weber T, Graf R et al. Efficient generation of Rosa26 knock-in mice using CRISPR/Cas9 in C57BL/6 zygotes. BMC Biotechnol 2016;16:4–18. 26772810 10.1186/s12896-016-0234-4 PMC4715285

[19] Hattori Y, Ogaki T, Ishimura M et al. Development and elucidation of a novel fluorescent boron-sensor for the analysis of Boronic acid-containing compounds. Sensors 2017;17:2436–42. 29064412 10.3390/s17102436 PMC5677422

[20] Hattori Y, Ishimura M, Ohta Y et al. Visualization of Boronic acid containing pharmaceuticals in live tumor cells using a fluorescent boronic acid sensor. ACS Sens 2016;1:1394–7.

[21] Hattori Y, Ishimura M, Ohta Y et al. Detection of boronic acid derivatives in cells using a fluorescent sensor. Org Biomol Chem 2015;13:6927–30. 26022725 10.1039/c5ob00753d

[22] Li B, Severson E, Pignon J-C et al. Comprehensive analyses of tumor immunity: implications for cancer immunotherapy. Genome Biol 2016;17:174. 27549193 10.1186/s13059-016-1028-7 PMC4993001

[23] Li T, Fan J, Wang B et al. TIMER: a web server for comprehensive analysis of tumor-infiltrating immune cells. Cancer Res 2017;77:e108–10. 29092952 10.1158/0008-5472.CAN-17-0307 PMC6042652

[24] Li T, Fu J, Zeng Z et al. TIMER2.0 for analysis of tumor-infiltrating immune cells. Nucleic Acids Res 2020;48:W509–14. 32442275 10.1093/nar/gkaa407 PMC7319575

[25] Houston KA, Henley SJ, Li J et al. Patterns in lung cancer incidence rates and trends by histologic type in the United States, 2004-2009. Lung Cancer 2014;86:22–8. 25172266 10.1016/j.lungcan.2014.08.001 PMC5823254

[26] Kawabata S, Miyatake S-I, Kuroiwa T et al. Boron neutron capture therapy for newly diagnosed glioblastoma. J Radiat Res 2009;50:51–60. 18957828 10.1269/jrr.08043

[27] Kawabata S, Suzuki M, Hirose K et al. Accelerator-based BNCT for patients with recurrent glioblastoma: a multicenter phase II study. Neurooncol Adv 2021;3:vdab067. 34151269 10.1093/noajnl/vdab067 PMC8209606

[28] Hanaoka K, Watabe T, Naka S et al. FBPA PET in boron neutron capture therapy for cancer: prediction of 10B concentration in the tumor and normal tissue in a rat xenograft model. EJNMMI Res 2014;4:70–7. 25621196 10.1186/s13550-014-0070-2 PMC4293470

[29] Watanabe T, Tanaka H, Fukutani S et al. L-phenylalanine preloading reduces the 10B(n, α)7Li dose to the normal brain by inhibiting the uptake of boronophenylalanine in boron neutron capture therapy for brain tumours. Cancer Lett 2016;370:27–32. 26455769 10.1016/j.canlet.2015.10.004

[30] Ohnishi K, Misawa M, Sikano N et al. Enhancement of cancer cell-killing effects of boron neutron capture therapy by manipulating the expression of L-type amino acid transporter 1. Radiat Res 2021;196:17–22. 33956158 10.1667/RADE-20-00214.1

[31] Wittig A, Sheu-Grabellus S-Y, Collette L et al. BPA uptake does not correlate with LAT1 and Ki67 expressions in tumor samples (results of EORTC trial 11001). Appl Radiat Isot 2011;69:1807–12. 21367608 10.1016/j.apradiso.2011.02.018

